# P052 Vestibulocochlear involvement in giant cell arteritis: expanding the clinical spectrum

**DOI:** 10.1093/rap/rkag113.082

**Published:** 2026-11-03

**Authors:** Hajra Ali Choudhry, Claire Wood

**Affiliations:** Glasgow Royal Infimary, Glasgow, United Kingdom; Glasgow Royal Infimary, Glasgow, United Kingdom

## Abstract

**Introduction:**

Giant cell arteritis (GCA) is a multisystemic granulomatous vasculitis seen in the elderly population that involves large and medium-sized blood vessels with predisposition for the cranial arteries. Classically, patients with GCA present with cranial ischemic manifestations that are directly related to vascular involvement. Although the typical non-ocular features of GCA are well known (e.g., new-onset headache, scalp tenderness, jaw claudication), vestibulocochlear presentations are rare and can mimic acute idiopathic sensorineural hearing loss (AISHL).

Here, we present an interesting case of a patient with sudden onset auditory and vestibular symptoms leading to biopsy proven GCA with presumed vestibulocochlear involvement.

**Case description:**

A 59-year-old woman, previously fit and well, developed sudden onset left-sided auditory and vestibular symptoms in October 2024. She described a sensation of left ear blockage, reduced hearing, tinnitus, vertigo, and imbalance. There was no preceding illness or other clear trigger. Several courses of antibiotics, for presumed ear infection, were given without symptomatic improvement.

Over the following weeks to months, she developed systemic symptoms including weight loss, fatigue, small joint discomfort, and progressive pain affecting her shoulders and upper arms associated with significant morning stiffness, prompting hospital admission.

Laboratory investigations demonstrated markedly raised ESR and CRP (both greater than 100), thrombocytosis, and normocytic anaemia. Further immunological testing showed a strongly positive rheumatoid factor, positive ANA, and anti-DNA antibodies by immunofluorescence, with negative anti-CCP antibodies, ENA, MPO and PR3 antibodies. There were no clinical features to suggest rheumatoid arthritis or a connective tissue disease.

CT head, MRI brain and inner ear were unremarkable. CT chest, abdomen and pelvis excluded malignancy but showed non-specific enlarged para-aortic lymph nodes.

On re-visiting the history she reported mild headache with abnormal sensation around the left temple and left side of her face. This had been more prominent at the time of onset of her auditory and vestibular symptoms and had improved somewhat by the time of her hospital admission.

Due to the combination of headache, polymyalgia rheumatica-like symptoms, raised inflammatory markers, and reduced left temporal artery pulsation on examination, giant cell arteritis (GCA) was suspected. A temporal artery biopsy was performed which demonstrated focal active arteritis with adventitial and transmural inflammation consistent with GCA.

She was commenced on high-dose prednisolone, initially 60 mg daily, with improvement in her headache and musculoskeletal symptoms. Tocilizumab was subsequently introduced alongside steroid tapering with continued improvement in most of her symptoms. Unfortunately her vestibulocochlear manifestations failed to completely settle.

**Discussion:**

GCA is a systemic large-vessel vasculitis affecting individuals over the age of 50. It commonly presents with headache, scalp tenderness, jaw claudication, visual symptoms, and polymyalgia rheumatica. This case highlights an unusual presentation of biopsy-proven GCA with prominent vestibulocochlear symptoms preceding the more classical manifestations of the disease.

The initial presentation of sudden unilateral hearing loss, tinnitus, vertigo, and imbalance created a diagnostic challenge. These symptoms prompted investigation for an ENT or neurological cause, with imaging failing to identify a structural explanation. Although audio vestibular manifestations are recognised complications of other systemic vasculitides, involvement of the vestibulocochlear nerve in GCA is uncommon and may be overlooked. The temporal association with systemic inflammatory symptoms and subsequent confirmation of GCA, and her clinical improvement with treatment suggests that these symptoms were likely related to vasculitic involvement.

Treatment followed current principles for GCA management, with urgent glucocorticoid therapy. Persistent vestibular and auditory symptoms despite steroid therapy led to the addition of tocilizumab, as a steroid-sparing agent. The patient’s inflammatory markers normalised during treatment, although symptoms did not completely resolve, illustrating the difficulty in distinguishing active vasculitis from residual neurological injury or alternative causes. A review of published case reports and a recent prospective study suggests that the vestibulocochlear of GCA, especially hearing loss, are often irreversible.

Interestingly, she also went on to develop a peripheral inflammatory arthritis necessitating a change from tocilizumab to methotrexate. Due to issues with deranged liver function tests methotrexate had to be stopped and she has recently been started on upadacitinib instead which should treat both the GCA and inflammatory arthritis.

This case demonstrates the importance of recognising atypical manifestations of GCA. Early diagnosis and treatment are essential to prevent complications, while persistent non-classical symptoms require ongoing multidisciplinary assessment.

**Key learning points:**

• Vestibulocochlear involvement is a rare presentation of GCA with only a handful of cases previously reported in the literature. As a result, it is easily misdiagnosed or missed altogether and is usually not considered when patients are assessed for GCA. This case highlights the importance of expanding our awareness of the clinical spectrum of possible GCA symptoms when reviewing patients presenting with atypical neurological and ENT manifestations that could be in keeping with GCA. Clinicians should be aware that GCA can mimic AISHL.

• The presence of concomitant sudden onset hearing loss, tinnitus, vertigo, and imbalance paired with a headache or other suggestive cranial or systemic symptoms should prompt consideration of GCA as a diagnosis and relevant investigations for this. TAB remains an important test that can be helpful in cases with diagnostic uncertainty.

• Normal imaging does not exclude vasculitis. CT and MRI did not identify a cause for the patient’s symptoms, demonstrating that inflammatory vascular disease may not always produce radiological abnormalities.

• This case emphasises that GCA has a broad clinical spectrum and that recognising uncommon presentations can help prevent delayed diagnosis and reduce the risk of serious complications. It is possible that a delay to diagnosis in these atypical cases may contribute to the lack of improvement in the audiovestibular impairment that has previously been observed.

